# Resident Knowledge of Colorectal Cancer Screening Assessed by Web-Based Survey

**DOI:** 10.14740/jocmr1610w

**Published:** 2014-02-06

**Authors:** Stuart Akerman, Scott L. Aronson, Maurice A. Cerulli, Meredith Akerman, Keith Sultan

**Affiliations:** a NSLIJ - Western Suffolk Gastroenterology, Bay Shore, NY, USA; b Hofstra - Northshore LIJ School of Medicine, Hempstead, NY, USA; c Department of Internal Medicine, Division of Gastroenterology, Robert Wood Johnson University Hospital, New Brunswick, NJ, USA; d Department of Internal Medicine, Division of Gastroenterology, Northshore Long Island Jewish Medical Center, Manhasset, NY, USA; e Feinstein Institute for Medical Research, Manhasset, NY, USA

**Keywords:** Resident education, Colorectal cancer, Preventive medicine

## Abstract

**Background:**

To evaluate resident knowledge of colorectal cancer (CRC) screening guidelines and to define areas requiring attention.

**Methods:**

A survey was created using three published guidelines for CRC screening. Program directors for internal medicine residency programs were contacted within the metro New York City area to have their residents participate.

**Results:**

Five programs participated, and 115 responses were recorded. For the appropriate testing and interval to screen for CRC, 61/115 residents identified flexible sigmoidoscopy every 5 years, 108/115 identified colonoscopy every 10 years, 16/115 identified double contrast barium enema (DCBE) every 5 years and only 12/115 thought CT-colography every 5 years was appropriate. Only 40/115 respondents appropriately identified fecal occult blood testing (FOBT) administered in the patient’s home annually, while fecal immunohistochemical testing (FIT) annually at home was identified by 8/115 residents.

**Conclusion:**

While most residents seem knowledgeable regarding CRC screening with colonoscopy, many deficiencies remain. FOBT for screening purposes remains undervalued, and confusion about administering the test persists. The distinction between screening and prevention needs further reinforcement.

## Introduction

While the overall incidence of cancer has decreased during the years 2000-2006, colorectal cancer (CRC) remains the third most common malignancy. It is the third leading cause of cancer-related mortality among men and women individually, and the second leading cause of cancer-related mortality overall [1]. The incidence and mortality of CRC have declined over the last 10 years with wider population screening, but there is still room for improvement. A particular focus has been on the lower rates of screening for minority groups such as African Americans, already noted to have higher rates of CRC-related mortality and poorer outcomes [2].

Resident outpatient practices account for the primary medical care of many underserved populations. Numerous studies [3-7] have looked at the role of physicians-in-training in health maintenance and screening. Ward et al performed resident interviews to investigate what they felt were barriers to screening for their own patients. While they were aware of some of the beliefs and/or misconceptions within the community, they were unaware of many other recognized barriers to screening within the minority populations such as embarrassment related to the procedure and preparation, cost of the procedure to the patient and access to care [7]. The provider turnover in a resident practice itself can compromise effective health maintenance and screening. Identification of a regular health care provider is a known positive correlate for screening, but providers change every few years in the resident practice. Some studies even estimate that as many as 50% of patients are lost to follow-up of their chronic medical conditions and screenings when resident physicians graduate and pass their patients on to new providers [8].

It is expected that as resident physicians advance in their training, they become more educated regarding colon cancer screening methods, and in turn more of their patients will be appropriately screened. The data on this subject however have been mixed. Gennarelli et al administered questionnaires to interns, residents and attending physicians with the intention of assessing knowledge of the American College of Gastroenterology guidelines for CRC screening, and found that while overall knowledge was poor, it improved by year of education [9]. Conversely, Wong et al measured performance outcomes in multiple screening categories over 3 years of training, and found that actual patient screening rates were similar across all years [10]. It is unclear if training regarding CRC screening is suboptimal, or if the guidelines themselves, which do not uniformly agree on appropriate screening ages, rates and modalities, may be partially to blame [11-15]. The most recent guidelines also make a distinction between cancer detection and prevention. Prevention in this context means a test with the potential to detect adenomatous polyps, the removal of which has been shown to decrease the risk of future malignancies [16]. This terminology is appropriate, but may add an additional element of confusion. The purpose of our study was to evaluate knowledge of CRC screening methods across 3 years of internal medicine training, to demonstrate if knowledge of screening improves with advancing post-graduate year (PGY) level, and to identify specific targets areas for training improvement.

## Methods

An 11-question survey was created in November 2010 using three of the more well-known published guidelines for CRC screening (ACG, USPSTF and ACS guidelines [11, 15, 17, 18]) and uploaded to a survey manager website (www.kwiksurveys.com) to be accessed anonymously in order to mitigate any potential bias. The ASGE guidelines were not included into the creation of our survey, as they focused more on colonoscopy and sigmoidoscopy, and did not make many recommendations regarding alternative screening methods. The ACP (American College of Physicians) drafted a guideline statement [17] subsequent to the administration of our survey, but much of their statement was derived from the same sources as our survey. The first question simply asked for the year of training, in order to create a subset for analysis by year of training. Respondents had the ability to answer with multiple correct choices for each subsequent question, reflecting the multiple options presented in the source guidelines. A copy of the survey form has been made available as an addendum to this manuscript.

**Figure 1:**
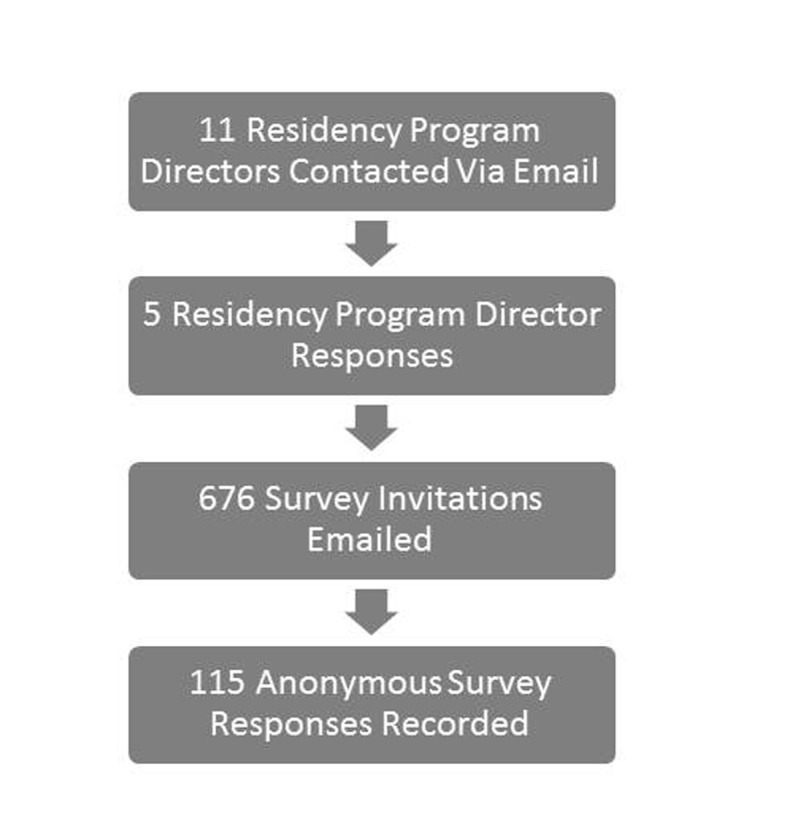
Survey enrollment.

Program directors for internal medicine residency programs at academic institutions were contacted within the metro New York City area, and asked to have their residents participate anonymously in the survey. Five programs agreed to participate, and 676 invitations were offered to their residents from December 2010 to January 2011. There were 115 responses, for a response rate of 17% (Fig. 1). All responses were anonymous and confidential, identified only by an I.P. address, and viewable only by the investigators. All responses were tabulated into a Microsoft Excel sheet and data were evaluated initially in aggregate, and then compared by PGY of training. All analyses were conducted using SAS version 9.3 (Cary, NC). The study was approved by the Institutional Review Board of the North Shore Long Island Jewish Health System.

## Results

The data were initially analyzed in aggregate, followed by a subset analysis. Of the 115 respondents, there were 53 PGY-1 residents, 32 PGY-2 residents and 30 PGY-3 residents. When polled regarding the appropriate testing and interval to screen for CRC, 61/115 residents identified flexible sigmoidoscopy every 5 years, 108/115 identified colonoscopy every 10 years, 16/115 identified double contrast barium enema (DCBE) every 5 years as an acceptable modality and interval and only 12/115 thought CT-colography (i.e. virtual colonoscopy) every 5 years was appropriate. When questioned regarding alternative methods, only 40/115 appropriately identified fecal occult blood testing (FOBT) administered in the patient’s home annually while fecal immunohistochemical testing (FIT) annually at home was identified by 8/115 residents. Fecal DNA testing was considered an option by only 7 residents, with 2 out of 7 agreeing with testing every 3 years, and the remaining 5 recommending an annual examination.

The questions were also readdressed for CRC prevention rather than screening/detection. Only 73/115 thought colonoscopy every 10 years was appropriate for cancer prevention, 44/115 flexible sigmoidoscopy every 5 years and 6/115 thought flexible sigmoidoscopy every 10 years was appropriate. Few respondents (10/115) found DCBE every 5 years to be appropriate, with a slightly larger number of respondents (15/115) identifying CT colography every 5 years. There were significantly fewer responses for FOBT, FIT and fecal DNA, but 18/115 incorrectly identified annual FOBT in the home, and 21/115 incorrectly identified annual office FOBT as viable options for CRC prevention.

Subset analysis also compared individuals based on their PGY of training (Table 1). All answers were compared using Chi-squared analysis, unless there were too few responses in any one instance, in which case Fisher’s exact testing was calculated. There was not a statistically significant difference in knowledge by year of training, except for annual home FOBT as a CRC detection test.

**Table 1:** Number of Responders Correctly Identifying Appropriate Exams for Detection of CRC and Their Associated Intervals

Screening exam	PGY-1	PGY-2	PGY-3	P value
Flexible sigmoidoscopy every 5 years	29 (54.72%)	17 (53.13%)	15 (50.00%)	0.9179
Colonoscopy every 10 years	49 (92.45%)	31 (96.88%)	28 (93.33%)	0.7852
Virtual colonoscopy every 5 years	5 (9.43%)	3 (9.38%)	4 (13.33)	0.8332
Annual FOBT performed in the patient’s home	12 (22.64%)	12 (37.50%)	16 (53.33%)	*0.0174
Annual FIT performed in the patient’s home	3 (5.66%)	1 (3.13%)	1 (3.33%)	1.0000
Fecal DNA testing every 3 years	0 (0.00%)	1 (3.13%)	1 (3.33%)	0.2885
DCBE every 5 years	9 (16.98%)	2 (6.25%)	5 (16.67%)	0.3370

*Denotes a statistically significant value.

Nearly all (112/115) of those polled were aware that 50 years of age is considered the appropriate time to begin screening for CRC in average risk adults, but only 18/115 recommended screening of average risk African Americans beginning at age 45, as mentioned in the current ACG practice guidelines [19]. For patients with first degree relatives with CRC before age 60, 76/115 respondents appropriately identified age 40 or 10 years younger than the index case as the appropriate time to begin screening for CRC.

For patients with first degree relatives diagnosed with CRC after age 60, or with multiple second degree relatives, responses varied. Forty out of 115 residents answered that 50 is an appropriate age to begin, no different than average risk screening, which is consistent with the ACG guidelines. Nine out of 115 respondents thought 40 years of age would be an appropriate age to begin screening, which is recommended by the current joint American Cancer Society/US Multi-Society Task Force/American College of Radiology guidelines.

Nearly all (114/115) respondents appropriately identified colonoscopy as a valid screening test for patients with first degree relatives with CRC before age 60. However, 33/115 incorrectly chose flexible sigmoidoscopy, 32/115 FOBT, 17/115 CT colography, 5/115 FIT, 5/115 fecal DNA and 7/115 DCBE.

Subset analysis of appropriate ages to begin screening in the various populations, evaluated by PGY of training can be found in Table 2.

**Table 2:** Responses to Appropriate Age to Begin Screening for CRC in Various Populations

When to begin screening?	PGY-1	PGY-2	PGY-3	P value
For average risk patients begin at age 50	52 (98.11%)	31 (96.88%)	29 (96.67%)	1.000
For average risk African-American patients begin at age 45	11 (20.75%)	3 (9.38%)	4 (13.33%)	0.3460
For patients with a first degree relative with CRC < age 60 begin at age 40 or 10 years younger than the index case	34 (64.15%)	20 (62.50%)	22 (73.33%)	0.6141
For patients with a first degree relative with CRC > age 60, or two second degree relatives with CRC begin at age 40^^	3 (5.66%)	3 (9.38%)	3 (10.00%)	0.7383
For patients with a first degree relative with CRC > age 60, or two second degree relatives with CRC begin at age 50^	12 (22.64%)	11 (34.38%)	14 (46.67%)	*0.0756

*Denotes a statistically significant value. ^Appropriate answer as per ACG 2008 guidelines. ^^Appropriate answer as per ACS 2008 guidelines.

## Discussion

While meant to guide, standardize, and simplify population wide colon cancer screening, the current, multiple guidelines themselves may be a source of confusion for healthcare providers, especially for those in training. While basic principles of the guidelines, such as screening at age 50 for average risk patients, are relatively uniform among all the guidelines, some of the other recommendations are conflicting (Table 3). For example, in regard to a patient with a first degree relative diagnosed with CRC after age 60 or two second degree relatives with CRC, the guidelines differ in their recommendations for the age to begin screening. The most recent ACG guidelines [11] recommend screening at the default age of 50, but the most recent American Cancer Society guidelines [15] advocate more aggressive screening by beginning at age 40.

**Table 3:** Initiation of Screening - Comparison of the Guidelines (ACS/USPSTF/ACG/ASGE)

When to begin screening?	ACS	USPSTF	ACG	ASGE
For average risk patients	Begin at age 50	Begin at age 50	Begin at age 50	Begin at age 50
For average risk African-American patients	Begin at age 45	Not addressed	Not addressed	Not addressed
For patients with a first degree relative with CRC < age 60	Begin at age 40 or 10 years younger than the index case	Not addressed	Begin at age 40 or 10 years younger than the index case	Begin at age 40 or 10 years younger than the index case
For patients with a first degree relative with CRC > age 60, or two second degree relatives with CRC	Begin at age 40	Not addressed	Begin at age 50 (same as average risk screening)	Begin at age 40

*Our study concept was designed with the ACS/USPSTF/ACG guidelines. The ASGE guidelines are provided for further comparison.

A national survey performed by Oxentenko et al [20] sought to evaluate how confident and prepared residents within primary care programs felt in regard to CRC guidelines and recommendation. Most of the residents polled responded that they were somewhat comfortable with the guidelines and their implementation, and their level of comfort seemed to increase by PGY. Our survey findings suggest that this comfort may be misplaced. The findings were favorable in regard to screening colonoscopy in most average risk individuals, but knowledge of other methods was more disparate, and did not improve with advancing years of education within the programs. Similarly a study published in 2005 sought to compare screening rates for patients of PGY1 and PGY2 residents for multiple preventative healthcare areas including CRC, to evaluate if “seniority” was a factor for improvement [21]. The authors found that there was no statistically significant difference in the screening rates between both years of training.

While our study was performed specifically in internal medicine residencies, it did not distinguish between those residents who were training under a traditional “categorical” track or those training in a “primary care” - focused track. An et al differentiated between the two groups when they evaluated performance for preventive screening tests in breast, cervical and colorectal cancers. The authors found that year of training did not seem to improve screening rates, nor did a specific primary care focus in the residents’ training [6].

Two points of particular interest become clear when reviewing the results of our study. First, there appears to be significant confusion among the resident physicians between the concepts of cancer detection and cancer prevention. The guidelines themselves lend to this confusion, as only the ACG and the ACS/US Multi-Society Task Force guidelines acknowledge differences in regard to patient testing; the ASGE guidelines do not. The US Preventive Services Task Force (USPSTF) omits any distinction between detection and prevention, while recommending certain tests such as colonoscopy, sigmoidoscopy and FOBT, and rejecting others such as fecal DNA or CT colography found in the other guidelines [18].

The second point highlighted by the survey is that alternative means for cancer detection beyond colonoscopy and sigmoidoscopy need to be better stressed during the education of our residents. This again is not uniformly agreed upon by the various guidelines (Table 4). There was an inadequate level of knowledge demonstrated by the respondents, with no improvement in knowledge by year, regarding any of the alternative screening modalities with the exception of annual home-administered FOBT. There was statistically significant improvement by year in regard to correct administration of FOBT, but still low recognition overall. There was a low recognition of the value of stool genetic testing and CT colography, but these may be deficient for the simple reason that neither is currently a routinely covered service by third party payers or Medicaid. This is certainly not the case however for an annual FOBT. We suspect that the strong current emphasis on colonoscopy-based screening may come at the expense of developing the knowledge and expertise for other modalities such as standard, proven FOBT. Residents may learn about the alternatives to colonoscopy from their textbooks, but only experience the alternatives in limited clinical practice.

**Table 4:** Screening Interval Comparison by Modality (ACS/USPSTF/ACG/ASGE)

	ACS	USPSTF	ACG	ASGE
Flexible sigmoidoscopy	Every 5 years	Not recommended	Every 5 - 10 years	Every 5 years
Colonoscopy	Every 10 years	Every 10 years	Every 10 years	Every 10 years
DCBE	Every 5 years	No recommendation given	No recommendation given	Not recommended, but if used, perform every 5 years
Virtual colonography	Every 5 years	No recommendation given	Every 5 years	Not recommended
FOBT	Annually	Annually	Annually	Annually
FIT	Annually	Annually	Annually	No recommendation given
Fecal DNA	Interval uncertain	No recommendation given	Every 3 years	Not recommended

*Our study concept was designed with the ACS/USPSTF/ACG guidelines. The ASGE guidelines are provided for further comparison.

While the rates of screening colonoscopies have increased in the last few years, there still remain patients who do not wish to have colonoscopies performed as a first-line examination due to the invasive nature of the test, as well as the preparation for the exam. Also, while the residents were surveyed in a community with ample access to colonoscopy services, many geographic regions lack this access. We need to better educate our providers so that they will be equipped to offer alternatives to colonoscopy when necessary, and therefore continue to increase the rate of screening among their patients.

While our focus has been upon improving physician education, we acknowledge that other solutions to improve CRC screening rates exist. Lane et al [22] showed that in a large county health center, continuing education initiatives with providers and their staff can increase internal completion rates for CRC screening, both for FOBT and colonoscopy. It is worth highlighting that the authors chose a two-pronged approach (administering an evidence-based lecture and applying a clinical prompt) to increase awareness and education regarding CRC screening. Education alone does not seem to be an adequate method to improve screening rates. Seres et al [23] evaluated two comparison initiatives at the provider level to improve the quality of care in CRC screening: an evidence-based lecture regarding screening guidelines, compared to the lecture in addition to a written prompt on the patient chart for all applicable patients. The study found that clinical prompts significantly improved completion of CRC screenings among physician providers when compared to administering evidence-based lectures alone. Another study performed by Schroy et al [24] compared the utility of providing screening education for resident physicians using didactic lectures in comparison to didactic lectures in combination with an interactive case-based model. They found that while there was a small, statistically significant improvement in screening rates for those residents who received both educational interventions, the authors felt that it still had limited influence on clinical risk assessment skills. These studies imply that while education is a valuable tool, there may be a need for additional interventions to improve screening performance.

### Conclusion

While most residents seem knowledgeable regarding CRC screening with colonoscopy and the proper screening age for average risk individuals, many deficiencies remain. FOBT for screening purposes remains undervalued, and confusion about properly administering the test persists. The distinction between screening and prevention, addressed in the ACG and US Multi-Society Task Force guidelines but absent from the USPSTF recommendations, needs further reinforcement. For patients with a high risk family history, there appears to be a knowledge deficit, though the discrepancies between the different guidelines may themselves contribute to the confusion. Based on our results we conclude that the education of medical residents regarding CRC screening and prevention guidelines is deficient, and we have identified clear and vital target areas for further reinforcement.

## Abbreviations

CRC: colorectal cancer
DCBE: double contrast barium enema
FOBT: fecal occult blood testing
FIT: fecal immunohistochemical testing
USPSTF: US Preventive Services Task Force
